# 16p13.3 duplication associated with non-syndromic pierre robin sequence with incomplete penetrance

**DOI:** 10.1186/s13039-014-0076-5

**Published:** 2014-11-25

**Authors:** Mingran Sun, Han Zhang, Guiying Li, Xianfu Wang, Xianglan Lu, Andrea Sternenberger, Carrie Guy, Wenfu Li, Jiyun Lee, Lei Zheng, Shibo Li

**Affiliations:** Department of Pediatrics, University of Oklahoma Health Sciences Center, Oklahoma City, OK 73104 USA; Key Laboratory for Molecular Enzymology and Engineering, College of Life Sciences, Jilin University, Changchun, Jilin 130012 P. R. China; Department of Obstetrics and Gynecology, Child and Family Research Institute, University of British Columbia, Vancouver, British Columbia V6T 1Z4 Canada; Department of Pathology, College of Medicine, Korea University, Seoul, 136-701 South Korea; Genetics Laboratory, Gansu Provincial Maternity and Child-Care Hospital, Lanzhou, Gansu 730050 P. R. China

**Keywords:** Small supernumerary marker chromosome, Pierre robin sequence, Array CGH, FISH, 16p13.3

## Abstract

**Background:**

Pierre Robin sequence (PRS) is a condition present at birth. It is characterized by micrognathia, cleft palate, upper airway obstruction, and feeding problems. Multiple etiologies including genetic defects have been documented in patients with syndromic, non-syndromic, and isolated PRS.

**Case presentation:**

We report a 4-year-old boy with a complex small supernumerary marker chromosome (sSMC) who had non-syndromic Pierre Robin sequence (PRS). The complex marker chromosome, der(14)t(14;16)(q11.2;p13.13), was initially identified by routine chromosomal analysis and subsequently characterized by array-comparative genomic hybridization (array CGH) and confirmed by fluorescence *in situ* hybridization (FISH). Clinical manifestations included micrognathia, U-type cleft palate, bilateral congenital ptosis, upslanted and small eyes, bilateral inguinal hernias, umbilical hernia, bilateral clubfoot, and short fingers and toes. To our best knowledge, this was the first case diagnosed with non-syndromic PRS associated with a complex sSMC, which involved a 3.8 Mb gain in the 14q11.2 region and an 11.8 Mb gain in the 16p13.13-pter region.

**Conclusions:**

We suggest that the duplicated chromosome segment 16p13.3 possibly may be responsible for the phenotypes of our case and also may be a candidate locus of non-syndromic PRS. The duplicated *CREBBP* gene within chromosome 16p13.3 is associated with incomplete penetrance regarding the mandible development anomalies. Further studies of similar cases are needed to support our findings.

## Background

The Pierre Robin sequence (PRS) is characterized by micrognathia, cleft palate, upper airway obstruction, and feeding problems [1]. The prevalence of PRS is estimated to be 1 in 8,500-14,000 births in the general population [2,3]. The ratio between male and female is equal [3,4]. Approximately 25% of PRS diagnosed in patients is associated with a known syndrome, 35% of patients have other abnormalities that do not constitute a recognizable syndrome (non-syndromic), and the remaining 40% of patients present with an isolated manifestation of PRS [5-7]. Although the first PRS case was described in 1923 [8], the pathogenic mechanism of PRS remains unclear. Embryologically, micrognathia is believed to be the primary defect which triggers a sequence of events including glossoptosis, with or without cleft palate. In theory, etiologies such as alcohol abuse during pregnancy, environmental factors, and hereditary factors resulting in micrognathia early in development can lead to PRS [9]. Mechanical obstructions including uterine compression, oligohydramnios, and fibroids may be related to abnormal mandibular growth [6,10-12]. Specific gene mutations and chromosomal anomalies including translocations, deletions, and duplications have also been documented in patients with syndromic, non-syndromic, and isolated PRS.

We report a boy with non-syndromic PRS due to a small supernumerary marker chromosome (sSMC). The sSMC was due to an unbalanced translocation between part of the long arm of chromosome 14 (14q11.2) and the terminal region of the short arm of chromosome 16 (16pter-p13.13), which led to duplications of these two chromosomal regions. To the best of our knowledge, this is the first report of a patient with PRS and a sSMC.

## Case presentation

The proband was the first child born to healthy non-consanguineous parents (maternal age 19 years) at a gestational age of 38 weeks. Physical examination at birth showed micrognathia, U-type cleft palate, bilateral congenital ptosis, upslanted and small eyes, bilateral inguinal hernias, umbilical hernia, bilateral clubfoot, and short fingers and toes. These features are consistent with clinical features of non-syndromic PRS. At the age of 4.5 months, he showed significant developmental delay. At 5 months old, he underwent bilateral inguinal hernia repair. At the age of 13 months, he had bilateral frontalis suspension surgeries. Five months later, he underwent a bilateral mandibular ramus osteotomy, a bilateral mandibular distraction using an external multiplanar device via intraoral incisions, and mandibular distraction device placement. At the age of 24 months, his clubfoot was treated with ponseti casting. The family history was noncontributory. His mother was not exposed to secondhand smoke or infectious diseases and denied alcohol or drugs abuse in pregnancy.

## Results

**Figure 1:**
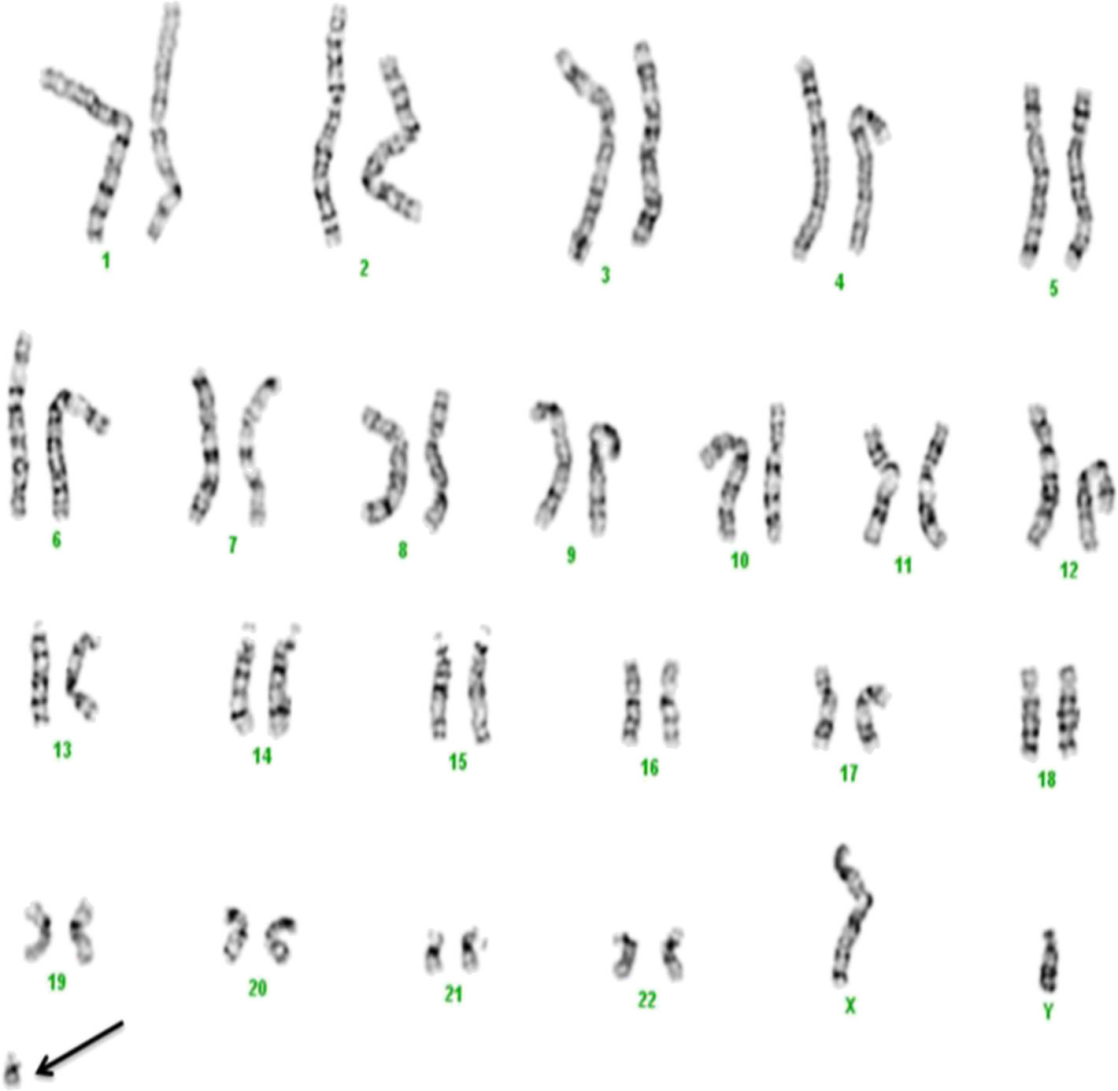
**Karyotype result.** G-banding revealed a karyotype 47, XY, +mar in all studied cells. The marker is highlighted by an arrow.

G-banding of chromosomes derived from the peripheral blood showed that each of the 21 cells analyzed had a modal number of 47 chromosomes including one X and one Y chromosome and a small supernumerary marker chromosome of unknown chromosomal origin. Based on the morphology, this marker chromosome appeared to be derived from one of the acrocentric chromosomes, but the size was smaller than chromosome 21 (Figure 1).

**Figure 2:**
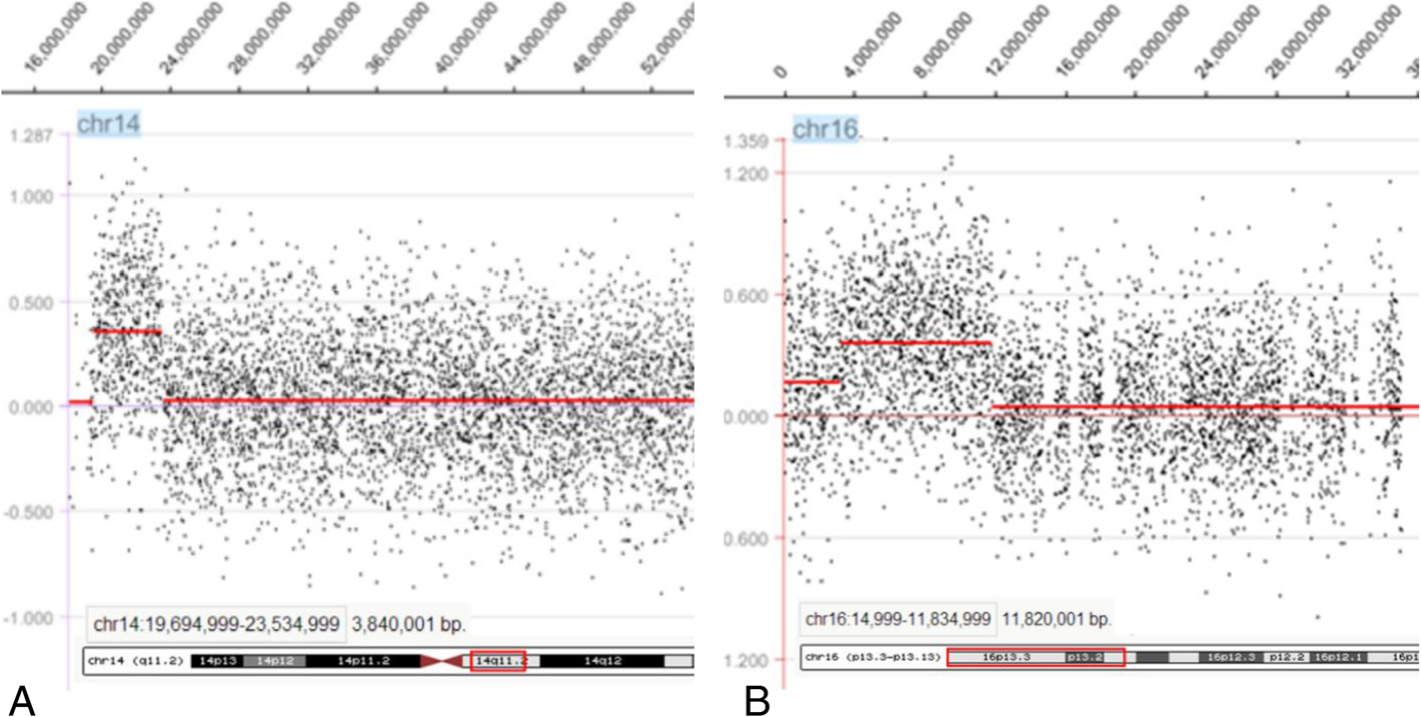
**Result of the** **array CGH genotyping (GRCh36/hg18). (A)** The sSMC of the patient characterized after array CGH covering 3.8 Mb [arr 14q11.2(19,694,999-23,534,999) × 3] in chromosome 14. **(B)** The sSMC of the patient characterized after array CGH covering 11.8 Mb [arr 16pterp13.13(14,999-11,834,999) × 3] in chromosome 16.

**Figure 3:**
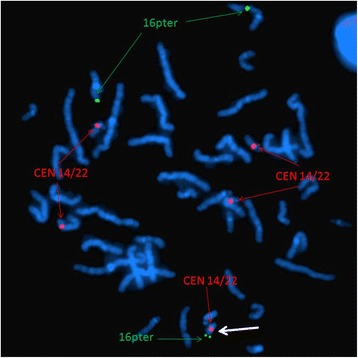
**Confirmatory FISH result.** Confirmatory FISH of this sSMC using centromere probe 14/22 (red) and TelVysion 16p (green) specific for 16pter revealed five distinct red signals and three distinct green signals confirming the origins of the sSMC. The marker is highlighted by a white arrow.

Array CGH is the current standard assay used to determine the chromosomal origin and the genomic size of non-mosaic chromosomal imbalances such as this case. Unexpectedly, array CGH showed two different chromosomal segment gains: the first was the gain of 14q11.2 region with 3.8 Mb (19,694,999-23,534,999) and the second one was the gain of 16p13.13-pter region with 11.8 Mb (14,999-11,834,999) (GRCh36/hg18, UCSC Genome Browser, February 2006 Assembly) (Figure 2). To confirm that the two chromosomal segments, 14q11.2 and 16pter-p13.13, were indeed joined together to form the marker chromosome, confirmatory FISH testing, utilizing the centromeric probe 14/22 (red) and the TelVysion 16p probe (green) specific for 16pter, was performed. All the metaphase cells analyzed showed that the marker chromosome had both the centromeric signal of chromosome 14/22 (red color) and the subtelomeric signal of 16pter (green color) indicating this marker chromosome was indeed derived from the unbalanced translocation between chromosomes 14 and 16, der(14)t(14;16)(q11.2;p13.13) (Figure 3).

## Discussion

To our best knowledge, this is the first report of a patient diagnosed with non-syndromic PRS associated with a complex sSMC, which involved chromosomes 14 and 16 at breakpoints of 14q11.2 and 16p13.13, respectively. Complex marker chromosome is one of the subgroups of sSMC which consists of chromosomal material derived from more than one chromosome and mostly associates with adverse clinical consequence [13]. Our patient is the second report case of a complex sSMC involving both chromosomes 14 and 16. However, the previously reported case involving both chromosomes 14 and 16 was der(14)t(14;16)(q12;q21), and the clinical features showed bilateral equinovarus, overlapping fingers, and intrauterine growth retardation, which were all different from the present case except the bilateral equinovarus [14]. Chromosomal anomalies are the third most common etiology in patients with PRS, occurring in 2% of all PRS cases [15]. Translocations involving 17q23-q25 are considered to be critical chromosomal regions that may be responsible for PRS based on sporadic case reports with t(2;17)(q23.3;q24.3), t(2;17)(q32;q24), t(5;17)(q15;q24), t(2;17)(q24.1;q24.3), and t(13;17)(q22.1;q23.3) [1,16,17]. The gene *SOX9,* located at chromosome 17q24 which was disrupted by translocation, was suggested to be responsible for non-syndromic PRS based on prior gene expression studies [16,17]. The sporadic cases with deletion of 2q32.3-q33.2, 4q25-q27, 4qter, 11q21-q23, 16q12.1-q13, and 22q12.2, and the duplication of 1q12-q25, 1q23.1-q31.1, 2q13-q21, 3q2, and 14qter were also reported in patients with PRS [1,4,18,19].

To investigate whether the duplicated 14q11.2 and pter-16p13.13 regions played a role in PRS development, all cases with comparable chromosome 14q and 16p duplication documented in the sSMC database [20] and PubMed were reviewed. A total of ten cases with sSMC at the breakpoint of 14q11.2 were identified in the database [20] (Table 1). In these ten sSMC cases, cytogenetically, three of them had ring-shaped sSMCs (cases 4, 7, and 8), three of them had minute-shaped sSMCs (cases 3, 9, and 10), two of them were dicentric (cases 1 and 2), and the remaining two were inverted duplicated sSMCs (cases 5 and 6). Mosaicism was presented in three cases (cases 2, 3, and 8). The identification and characterization methods of sSMCs included FISH, array CGH, and multiplex ligation-dependent probe amplification (MLPA). Six cases were characterized by FISH methods including cen-FISH, multiplex fluorescence *in situ* hybridization (M-FISH), and various FISH probes (cases 1, 2, 3, 5, 8, and 9). Three cases were investigated by array CGH (cases 5, 7, and 10). Multiplex ligation-dependent probe amplification was performed on one case (case 6). One case was studied by routine cytogenetic method (case 4). Clinically, cases 1, 2, 3, and 4 were reported to have normal phenotype. Case 5 and 6 were detected prenatally, with maternal inherited inv dup(14)(q11.2). The pregnancy outcomes of case 5 and 6 were unknown, but mothers with same markers were phenotypically normal. Thus, these six sSMCs do not appear to have any clinical phenotype. The remaining four cases, cases 7–10, showed variable clinical features. Both case 7 and case 8 had ring chromosome with similar breakpoints, r(14)(::p11.2 → q11.2::), however, the phenotypes of these two cases were different. Case 7 had hypogonadotopic hypogonadism, precocious puberty, small hands, scoliosis, hypogonadism, and moderate mental development delay, whereas case 8 only had dwarfism. As expected, the consequences of ring marker chromosomes were more complicated than other types of chromosomal anomalies. Clinical phenotypes of ring chromosomes are influenced by the size of the ring chromosome, rearrangement of the ring chromosome due to ring instability, and epigenetic factors caused by the circular architecture of ring chromosomes [21]. It is evident that because there were no overlapping phenotypes observed in cases 7 and 8, the clinical features were more likely related to ring chromosome complicated consequences other than dosage effects of the duplicated regions. Case 9 had a minute chromosome derived from chromosome 14 complicated with maternal UPD 14 and the patient had classical features of maternal UPD 14, i.e. microcephaly, developmental delay, small stature, and hypotonia [22]. In case 10, the patient only showed dysmorphic features. None of the above reported cases had micrognathia, which was the primary event in PRS, suggesting that chromosome 14q11.2 duplication was unlikely to be responsible for the PRS of our patient. Thus, chromosome 14q11.2 duplication does not appear to be a severe pathogenic copy number variation (CNV).

**Table 1:** **Clinical manifestations of the patients with sSMCs including region 14q11.2**

**Case # (no. in database)**	**de novo/inherited**	**Test methods**	**Final result of the sSMC**	**Clinical symptoms**
1 (14-O-q11.2/1-1)	de novo	cep 14/22; cep 15; cep 13/21	47,XY,+dic(14;15)(14pter → 14q11.2::15q11.1 → 15pter) [100%]	Normal
2 (14-O-q11.2/2-1)	n.a.	M-FISH; subcenM-FISH	47,XY,+dic(14)(:p11.1 → q11.1::p11.1 → q11.2:)[15]/46,XY[10]	Normal
3 (14-O-q11.2/3-1)	n.a.	acrocenM-FISH; subcenM-FISH	47,XX,+min(14)(pter → q11.2:)[40]/46,XX[60]	Normal female, fertility problems
4 (14-O-q11.2/4-1)	n.a.	n.a.	47,XY,+r(14)(::p11.2 → q11.2::)[100%]	Normal male, fertility problems - oligospermia
5 (14-O-q11.2/5-1)	maternal	acrocenM-FISH; subcenM-FISH; Array CGH	47,XY,+inv dup(14)(q11.2)[100%](1.79 MB)	Pregnancy outcome unknown but mother clinically normal
6 (14-O-q11.2/5-2)	maternal	MLPA	47,XY,+inv dup(14)(q11.2)[100%]	Abnormal first trimester screening; mother normal
7 (14-W-q11.2/2-1)	n.a.	Array CGH	47,XY,+r(14)(::p11.2 → q11.2::)[100%] (array-CGH data not available)	Hypogonadotopic hypogonadism, moderate mental developmental delay, precocious puberty, small hands, scoliosis
8 (14-W-q11.2/2-2)	n.a.	cenM-FISH; subcenM-FISH	47,XY,+r(14)(::p11.?2 → q11.2::)[14]/46,XY[16]	Normal, apart from dwarphism
9 (14-U-16)	de novo	Different FISH-probes; subcenM-FISH; UPD-test	47,XY,+min(14)(pter → q11.1 ~ q11.2:)[100%] maternal UPD 14	No mental retardation; at 4 y all values <3rd centile (height 85 cm, weight 11.5 kg, OFC 47,5 cm); microcephaly, simian crease, developmental delay, small stature, hypotonic
10 (14-W-q11.2/1-1)	n.a.	Array CGH	47,+min(14)(pter → q11.2)[100%] (20.17 MB)	Dysmorphic features

*n.a* not available.

**Figure 4:**
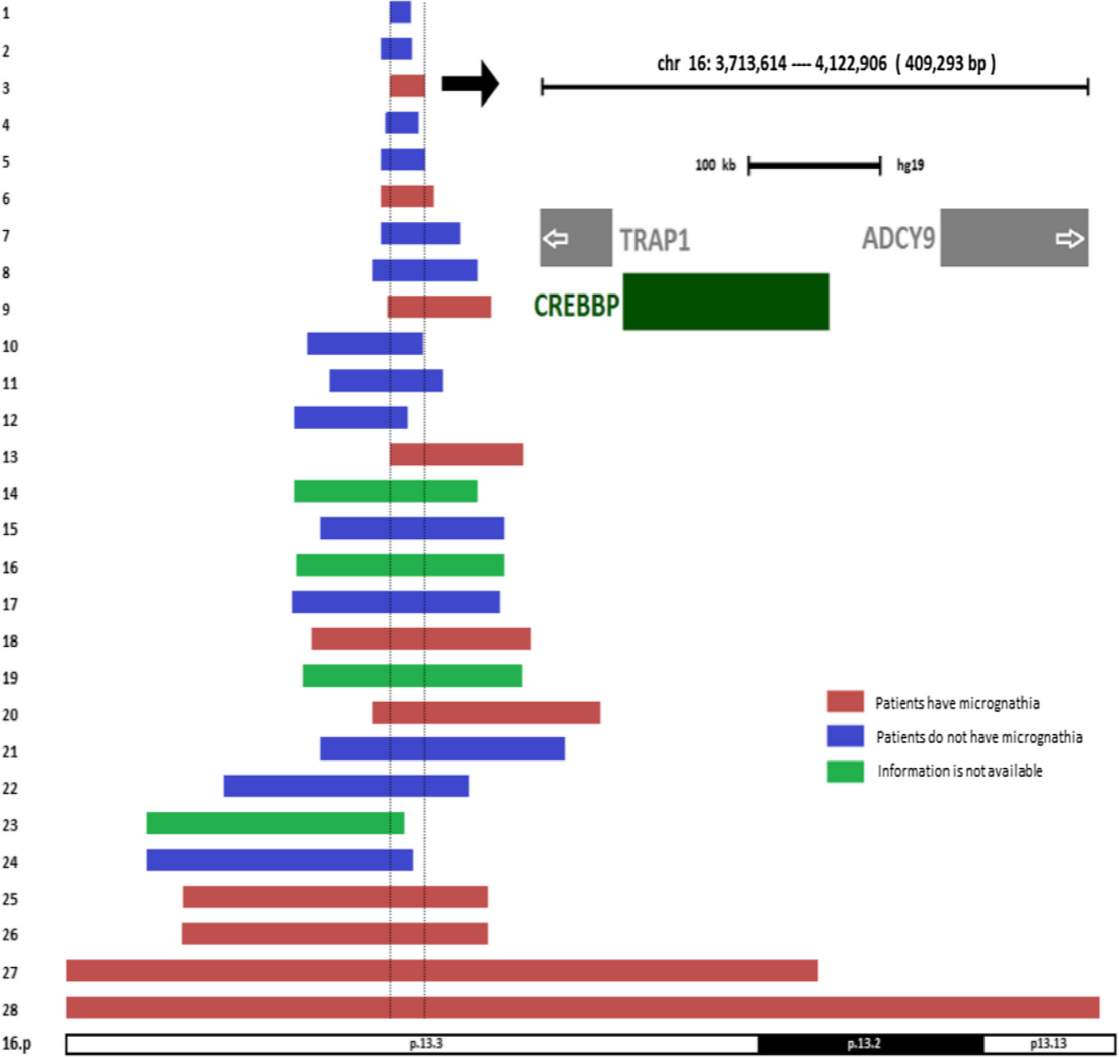
**Map of the chromosome 16p duplicated region.** The order of the cases follows the same order in Table 2.

Chromosomal 16p is a common region for chromosomal rearrangements. Twenty-seven cases with pure 16p duplication characterized by array CGH or single nucleotide polymorphism (SNP) array assay were found in the literature and summarized in Table 2. Cytogenetically, twenty-six of them only involved band 16p13.3, and the remaining one involved the 16p13.2-16pter region, whereas our case had a gain of 16p13.13-pter region (Figure 4). Phenotypically, close review identified that in those twenty-seven cases, plus our case, twenty-four of them had documented mandible development and ten had micrognathia. The remaining four cases did not have mandible developmental description (Table 2, Figure 4). Based on current evidence, genomic microduplicaion syndromes are frequently associated with incomplete penetrance and variable expression of phenotypes [23]. In this cohort, ten out of twenty-four cases developed micrognathia, indicating that chromosome 16p duplication is the cause of micrognathia, although the penetrance was not complete. Etiologically, any factors resulting in micrognathia in the early development could lead to PRS. However, abnormal mandible growth early in development does not instantly trigger the subsequent events, such as cleft palate and/or glossoptosis. Some patients might have the isolated small jaw or cleft palate without the airway obstruction. Thus, in those patients who had micrognathia, a smaller number of patients with cleft palate was observed, and only our patient had both cleft palate and airway obstruction and was diagnosed with PRS. Previous studies documented that genetically, the specific gene mutations, translocations, deletions, and duplications involving multiple chromosomes related to PRS [1,4,16-19]. This evidence supported that PRS was a disease with genetic heterogeneity. The chromosomal or genetic anomalies were the initiating events of the whole PRS sequence. Therefore, the duplicated 16p13.13-pter region likely led to micrognathia and was also responsible for the PRS phenotype of our patient. At this time, the underlying causes promoting small mandible into PRS are not clarified.

**Table 2:** **Clinical manifestations of the patients with partial 16p duplication**

**Clinical features**	**1**	**2**	**3**	**4**	**5**	**6**	**7**	**8**	**9**	**10**	**11**	**12**	**13**	**14**	**15**	**16**	**17**	**18**	**19**	**20**	**21**	**22**	**23**	**24**	**25**	**26**	**27**	**28**
	**Patient 1** [24]	**Patient 9** [25]	[26]	**Patient 2** [24]	**Patient 10** [25]	**Patient 11** [25]	**Patient 12** [25]	**Patient 8** [25]	**Patient 3** [24]	**Patient 4** [24]	**Patient 6** [25]	**Patient 2** [25]	**Patient 5** [24]	[27]	**Patient 5** [25]	[28]	**Patient 6** [24]	**Patient 3** [25]	[29]	**Patient 7** [25]	**Patient 4** [25]	**Patient 1** [25]	[30]	**Patient 7** [24]	**Patient 8** [24]	**Patient 9** [24]	[31]	**Present case**
Mental retardation	+	+	+	+	+	+	-	+		+	+	+	+	+	+	+	+	+	+	+	+	-	+	+	+	+	+	
Eye anomalies	+		+	+		+	+	+	+	+	+	+	-	+	+	+	+	+	+	+	+	+	+	+	+	+		+
Ptosis	+		+	-		+	-	-	-	+	-	+	-	+	-	+	+	+	-	-	+	-	+	-	+	+		+
Upslanting palpebral fissures	+		+	+		+	+	+	+	+	-	+	-	+	+	-	-	-	+	+	-	+	+	+	+	+		+
Short palpebral fissures	+		+	+		+	-	-	+	+	-	-	-	+	+	+	+	-	+	-	+	+	+	+	+	+		+
Nose anomalies	-	+	+	+	+	+	+	+	+	+	+	+	+	+	+	+	+	-	+	-	-	-		+	+	+	+	-
Cleft palate	-	-	-	-	-	-	-	-	+	-	-	-	+	+	-		-	-	-	-	-	-		-	+	+	+	+
Ears anomalies	-	+	+	+	+	+	+	+	+		+	+	+	+	+	+	+	-	+	+	+	-	+	+	+	+	+	-
Microcephaly	+	+	+	+	-	+	+	-	+	+	-		-	+			+					-		+	+	+	+	-
Long face/round face		-												+					+		+					+	+	-
Micrognathia	-	-	+	-	-	+	-	-	+	-	-	-	+		-		-	+		+	-	-		-	+	+	+	+
Bilateral inguinal hernia	+	-		+		-	+	+	-	-	-	+	-		-		-	-		-	-	-		-	+	-		+
Hand anomalies	+	+	+	+	+	+	+	+	+	+		+	+	+	+	+	+	+	+	+	+	+	-	+	+	+		+
Foot abnormality	+	-	+	+				+	-	+		+	-	+	+	+	+	+	-	+	+	+	+	+	+	-	+	+
Congenital heart disease	-	-	+	+	+	+		-	-	-	+	-	-	+		+	-	-	-	-	-	+		-	-	-	-	-
Break point (Mb)	3.7-3.9	3.6-3.9	3.7-4.1	3.6-4.0	3.6-4.0	3.6-4.2	2.6-4.5	3.5-4.7	3.7-4.9	2.8-4.1	3.0-4.3	2.6-3.9	3.7-5.2	2.6-4.7	2.9-5.0	2.6-5.0	2.6-5.0	2.8-5.3	2.7-5.2	3.5-6.1	2.9-5.7	1.8-4.6	0.9-3.8	0.9-3.9	1.3-4.8	1.3-4.8	0-8.6	0-11.8
Size (Mb)	0.24	0.35	0.41	0.47	0.5	0.6	0.9	1.2	1.2	1.3	1.3	1.3	1.52	2.1	2.1	2.37	2.4	2.5	2.5	2.6	2.8	2.8	2.9	3.04	3.5	3.5	8.6	11.8

Molecularly, in these nine cases, plus our case, with micrognathia, the array data showed that the size of the duplicated segments ranged from 0.409 Mb to 11.8 Mb. The overlapping region of those ten cases was approximately 0.409 Mb in size (Table 2, Figure 4). There were three genes mapped in this region including *TRAP1, CREBBP,* and *ADCY9*. The mutant *CREBBP* gene (OMIM 600140), or microdeletion of chromosome 16p13.3 including the *CREBBP* gene, is responsible for the development of the Rubinstein–Taybi syndrome (OMIM 180849) [32]. The significance of both *TRAP1* (OMIM 606219) and *ADCY9* (OMIM 603302) were unknown based on current evidence. Previous studies suggested that the *CREBBP* gene was dosage sensitive, and also responsible for the phenotype of chromosome 16p13.3 duplication syndrome [24-26]. Chromosome 16p13.3 duplication syndrome was due to the duplication of chromosome 16p13.3 encompassing the *CREBBP* gene and characterized by frequent clinical findings such as mild to middle intellectual disability, facial dysmorphism, anomalies of the extremities, and occasional developmental defect of the eyes, palate, genitalia, and heart (OMIM 613458). Both the genotype and phenotype of our case overlapped with chromosome 16p13.3 duplication syndrome, suggesting that the duplication of 16p13.3 was the pathogenic CNV in our case, and the *CREBBP* gene was the most critical candidate gene responsible for the phenotype of our patient. On the other hand, the overlapping region of all twenty-seven cases plus our case was 163 Kb in size (chr16:3,701,913-3,864,938). There were three genes mapped in this region including *DNASE1*, *TRAP1*, and *CREBBP*. The mutant *DNASE1* gene (OMIM 125505) was susceptible to systemic lupus erythematosus, which had no overlapping phenotype with presented cases. The significance of *TRAP1* (OMIM 606219) was unknown. The *CREBBP* gene was the most critical gene responsible for the phenotype based on current evidence [24-26]. For the twenty-eight cases listed in Table 2, all of them encompassed the *CREBBP* gene, and only ten of them had micrognathia. Thus, our literature review suggested that the duplicated *CREBBP* gene was associated with incomplete penetrance regarding the mandible development anomalies.

## Conclusion

In conclusion, our data and literature review suggest that the duplicated chromosome segment 16p13.3 may be responsible for the phenotype of the case presented and also may be a candidate locus of non-syndromic PRS. It is also noteworthy that the duplicated *CREBBP* gene was associated with incomplete penetrance regarding the mandible development anomalies. Further studies of similar cases are needed to support our findings.

## Materials and methods

At the age of two months, the proband was referred to us for cytogenetic evaluation. Cultures of the patient’s peripheral blood were established and harvested according to our standard laboratory protocols. Chromosome preparations were treated with trypsin and stained with Giemsa. A total of 21 metaphase cells were analyzed at 550-band resolution level. The karyotypes were described according to the guideline of An International System for Human Cytogenetic Nomenclature [33]. The parents declined to undergo chromosomal analysis.

A subsequent array-comparative genomic hybridization (array CGH) test was performed to determine the chromosomal origin of the sSMC, as well as other possible chromosome abnormalities which may have been missed by routine G-banded chromosomal analysis. Genomic DNA was extracted from the patient’s peripheral white blood cells using Nucleic Acid Isolation System (QuickGene-610 L, FUJIFILM Corporation, Tokyo, Japan) according to the manufacturer’s protocols. Human reference genomic DNA was purchased from Promega (Promega Corporation, Madison, WI, USA). The patient’s DNA and the purchased reference DNA were labeled with either cyanine 3 (Cy-3) or cyanine 5 (Cy-5) by random priming (Trilink Biotechnologies, San Diego, CA, USA). These samples were subsequently hybridized to a NimbleGen high-capacity 3 × 720 K oligo microarray chip (Roche/NimbleGen System Inc., Madison, WI, USA) by incubating in a MAUI Hybridization System (BioMicro Systems, Salt Lake City, UT, USA) for 40 hours according to NimbleGen’s CGH protocols. The array was scanned at 532 nm and 635 nm using the GenePix scanner (Molecular Devices, Sunnyvale, CA, USA). NimbleScan and SignalMap (NimbleGen System Inc, Madison, WI, USA) were applied for data analysis.

To verify array CGH findings, fluorescence *in situ* hybridization (FISH) was performed using the metaphase cells from the cell pellet. Two probes, the combination centromeric probe 14/22 and the TelVysion 16p probe specific for the terminal end of 16p (purchased from Abbott Corporation, Abbott Park, IL, USA) were used. Metaphase FISH analyses were performed according to our standard laboratory procedures. A total of 50 metaphase cells were analyzed.

## Consent

Since the patient information is completely de-identified and samples were received following the standard of care clinical evaluation, no IRB/consent is necessary based on the policy of the University of Oklahoma.

## Abbreviations

CGH: Comparative genomic hybridization
CNV: Copy number variation
FISH: Fluorescence *in situ* hybridization
PRS: Pierre robin sequence
sSMC: Small supernumerary marker chromosome
SNP: Single nucleotide polymorphism

## References

[1] Olasoji HO, Ambe PJ, Adesina OA (2007). Pierre robin syndrome: an update. Niger Postgrad Med J.

[2] Bush PG, Williams AJ (1983). Incidence of the robin anomalad (pierre robin syndrome). Br J Plast Surg.

[3] Printzlau A, Andersen M (2004). Pierre robin sequence in Denmark: a retrospective population-based epidemiological study. Cleft Palate Craniofac J.

[4] Jakobsen LP, Knudsen MA, Lespinasse J, Garcia Ayuso C, Ramos C, Fryns JP, Bugge M, Tommerup N (2006). The genetic basis of the pierre robin sequence. Cleft Palate Craniofac J.

[5] Marques IL, Barbieri MA, Bettiol H (1998). Etiopathogenesis of isolated robin sequence. Cleft Palate Craniofac J.

[6] Holder-Espinasse M, Abadie V, Cormier-Daire V, Beyler C, Manach Y, Munnich A, Lyonnet S, Couly G, Amiel J (2001). Pierre robin sequence: a series of 117 consecutive cases. J Pediatr.

[7] Al Kaissi A, Ganger R, Klaushofer K, Grill F (2011). Cervico-thoracic kyphosis in a girl with Pierre Robin sequence. Ger Med Sci.

[8] Robin P (1923). La chute de la base de la langue consideree comme une nouvelle cause de gene dans la respiration naso- pharyngienne. Bull Acad Med.

[9] Shprintzen RJ (1992). The implications of the diagnosis of robin sequence. Cleft Palate Craniofac J.

[10] Cohen MM (1976). The robin anomalad - its nonspecificity and associated syndromes. J Oral Surg.

[11] Taylor MR (2001). The pierre robin sequence: a concise review for the practicing pediatrician. Pediatr Rev.

[12] Aggarwal S, Kumar A (2003). Fetal hydrocolpos leading to pierre robin sequence: an unreported effect of oligohydramnios sequence. J Perinatol.

[13] Liehr T, Cirkovic S, Lalic T, Guc-Scekic M, de Almeida C, Weimer J, Iourov I, Melaragno MI, Guilherme RS, Stefanou EG, Aktas D, Kreskowski K, Klein E, Ziegler M, Kosyakova N, Volleth M, Hamid A (2013). Complex small supernumerary marker chromosomes - an update. Mol Cytogenet.

[14] Van Opstal D, Boter M, Noomen P, Srebniak M, Hamers G, Galjaard RH (2011). Multiplex ligation dependent probe amplification (MLPA) for rapid distinction between unique sequence positive and negative marker chromosomes in prenatal diagnosis. Mol Cytogenet.

[15] Izumi K, Konczal LL, Mitchell AL, Jones MC (2012). Underlying genetic diagnosis of pierre robin sequence: retrospective chart review at two children’s hospitals and a systematic literature review. J Pediatr.

[16] Benko S, Fantes JA, Amiel J, Kleinjan DJ, Thomas S, Ramsay J, Jamshidi N, Essafi A, Heaney S, Gordon CT, McBride D, Golzio C, Fisher M, Perry P, Abadie V, Ayuso C, Holder-Espinasse M, Kilpatrick N, Lees M, Picard A, Temple I, Thomas P, Vazquez MP, Vekemans M, Crollius HR, Hastie N, Munnich A, Etchevers H, Pelet A, Farlie P (2009). Highly conserved ono-coding elements on either side of SOX9 associated with pierre robin sequence. Nat Genet.

[17] Jakobsen LP, Ullmann R, Christensen SB, Jensen KE, Mølsted K, Henriksen KF, Hansen C, Knudsen MA, Larsen LA, Tommerup N, Tümer Z (2007). Pierre robin sequence may be caused by dysregulation of SOX9 and KCNJ2. J Med Genet.

[18] Gerard-Blanluet M, Pipiras E, Levaillant JM, Joye N, Koubi V, Kanafani S, Vergnaud A, Verloes A, Gonzales M, Jeny R, Benzacken B (2007). Prenatal detection of pierre robin sequence with deletion Xp and additional trisomy 14q by telomere screening. Prenat Diagn.

[19] Davidson TB, Sanchez-Lara PA, Randolph LM, Krieger MD, Wu SQ, Panigrahy A, Shimada H, Erdreich-Epstein A (2012). Microdeletion del(22)(q12.2) encompassing the facial development- associated gene, MN1 (meningioma 1) in a child with pierre-robin sequence (including cleft palate) and neurofibromatosis 2 (NF2): a case report and review of the literature. BMC Med Genet.

[20] Liehr T: **Small supernumerary marker chromosomes.** http://ssmc-tl.com/sSMC.html. [accessed 07/29/2014] 10.1186/1755-8166-7-S1-I11 PMC4043994 24940369

[21] Guilherme RS, Meloni VF, Kim CA, Pellegrino R, Takeno SS, Spinner NB, Conlin LK, Christofolini DM, Kulikowski LD, Melaragno MI (2011). Mechanisms of ring chromosome formation, ring instability and clinical consequences. BMC Med Genet.

[22] Mitter D, Buiting K, von Eggeling F, Kuechler A, Liehr T, Mau-Holzmann UA, Prott EC, Wieczorek D, Gillessen-Kaesbach G (2006). Is there a higher incidence of maternal uniparental disomy 14 [upd(14)mat]? Detection of 10 new patients by methylation-specific PCR. Am J Med Genet A.

[23] Berg JS, Potocki L, Bacino CA (2010). Common recurrent microduplication syndromes: diagnosis and management in clinical practice. Am J Med Genet A.

[24] Demeer B, Andrieux J, Receveur A, Morin G, Petit F, Julia S, Plessis G, Martin-Coignard D, Delobel B, Firth HV, Thuresson AC, Lanco Dosen S, Sjörs K, Le Caignec C, Devriendt K, Mathieu-Dramard M (2013). Duplication 16p13.3 and the CREBBP gene: confirmation of the phenotype. Eur J Med Genet.

[25] Thienpont B, Béna F, Breckpot J, Philip N, Menten B, Van Esch H, Scalais E, Salamone JM, Fong CT, Kussmann JL, Grange DK, Gorski JL, Zahir F, Yong SL, Morris MM, Gimelli S, Fryns JP, Mortier G, Friedman JM, Villard L, Bottani A, Vermeesch JR, Cheung SW, Devriendt K (2010). Duplications of the critical rubinstein–taybi deletion region on chromosome 16p13.3 cause a novel recognisable syndrome. J Med Genet.

[26] Mattina T, Palumbo O, Stallone R, Pulvirenti RM, Di Dio L, Pavone P, Carella M, Pavone L (2012). Interstitial 16p13.3 microduplication: case report and critical review of genotype-phenotype correlation. Eur J Med Genet.

[27] Tüysüz B, van Bon BW, Alp Z, Güzel Z, Veltman JA, de Vries BB (2012). A microduplication of the rubinstein-taybi region on 16p13.3 in a girl with a bilateral complete cleft lip and palate and severe mental retardation. Clin Dysmorphol.

[28] Chen JL, Yang YF, Huang C, Wang J, Yang Z, Tan Z (2012). Clinical and molecular delineation of 16p13.3 duplication in a patient with congenital heart defect and multiple congenital anomalies. Am J Med Genet A.

[29] Dallapiccola B, Bernardini L, Novelli A, Mingarelli R (2009). Expanding the phenotype of duplication of the rubinstein–taybi region on 16p13.3. Am J Med Genet.

[30] Friedman JM, Baross A, Delaney AD, Ally A, Arbour L, Asano J, Bailey DK, Barber S, Birch P, Brown-John M, Cao M, Chan S, Charest DL, Farnoud N, Fernandes N, Flibotte S, Go A, Gibson WT, Holt RA, Jones SJ, Kennedy GC, Krzywinski M, Langlois S, Li HI, McGillivray BC, Nayar T, Pugh TJ, Rajcan-Separovic E, Schein JE, Schnerch A (2006). Oligonucleotide microarray analysis of genomic imbalance in children with mental retardation. Am J Hum Genet.

[31] de Ravel T, Aerssens P, Vermeesch JR, Fryns JP (2005). Trisomy of chromosome 16p13.3 due to an unbalanced insertional translocation into chromosome 22p13. Eur J Med Genet.

[32] Hennekam RC (2006). Rubinstein-taybi syndrome. Europ J Hum Genet.

[33] Shaffer LG, McGowan-Jordan J, Schmid M (2013). An international system for human cytogenetic nomenclature.

